# Inverted Uterus Reduced after Four and a Half Years’ Duration

**Published:** 1869-04

**Authors:** 


INVERTED UTERUS REDUCED AFTER FOUR AND A HALF YEARS' DURATION.—Dr. A. K. Reynolds *(Humboldt Medical Archives)* reports that he was called to a patient in whom inversion of the uterus had existed for four and a half years. She had been attended by a midwife, who immediately after her confinement had put the patient upon her feet and pulled upon the adherent placenta until the uterus was completely inverted and protruded externally. A physician came, removed the placenta, and returned the uterus within the vagina, but failed to reduce the inversion. Subsequently it was treated by several physicians, some of whom cauterized the surface, supposing it to be ulceration of the cervix. Dr. Reynolds attempted to reduce the inversion, but after an hour's trial it was found necessary to abandon the operation until the rigidity of the cervix could be overcome. To overcome this an elastic air bag was introduced and inflated. By this means the parts were relaxed and the rigidity of the cervix in a great measure disappeared. A second attempt at reduction was successful in about twenty minutes. The patient made a perfect recovery.—*Medical Record.*

